# Analysis of HIV contact tracing in Yunnan Province: data-driven insights for optimizing HIV prevention and control strategies

**DOI:** 10.3389/fpubh.2025.1689070

**Published:** 2026-01-12

**Authors:** Yuhua Shi, Yu Han, Junli Huo, Huichao Chen, Xiaojing An, Xiaomei Jin, Zhimin Yang, Nuoya Xu, Xiao Zhang, Min Chen, Manhong Jia

**Affiliations:** 1 Institute for AIDS/STD Control and Prevention, Yunnan Center for Disease Control and Prevention (Yunnan Academy of Preventive Medicine), Kunming, Yunnan, China; 2 School of Public Health, Kunming Medicine University, Kunming, Yunnan, China; 3 Yunnan International Joint Laboratory of Public Health and Disease Control, Yunnan Center for Disease Control and Prevention (Yunnan Academy of Preventive Medicine), Kunming, Yunnan, China; 4 Yunnan Provincial Key Laboratory of Public Health and Biosafety, Yunnan Center for Disease Control and Prevention (Yunnan Academy of Preventive Medicine), Kunming, Yunnan, China

**Keywords:** China, contact tracing, HIV, recent infection, Yunnan

## Abstract

**Background:**

To effectively control the spread of HIV, this study evaluated contact tracing implementation among newly diagnosed individuals in Yunnan Province, China—a region heavily affected by HIV.

**Methods:**

From January to June 2020, contact tracing targeted newly diagnosed HIV cases to identify sexual/drug-injecting partners. Factors correlated with index cases, close contacts, and positive testing outcomes were analyzed.

**Results:**

Among 5,695 new HIV cases, 1,612 (28.3%) received tracing counseling, revealing 2,226 close contacts. Of the 1,612 index cases, 182 were recently infected, while 1,430 had been infected long-term. Of the 2,226 close contacts, 910 underwent HIV antibody testing, with 17.8% (162/910) testing positive. Notably, 132 of these contacts were newly diagnosed with HIV, representing 14.5% of those tested. The detection rates for both positive and newly diagnosed infections were higher among contacts identified by recently infected index cases than among those identified by long-term infected index cases. A multivariate analysis showed that HIV index cases of other ethnicities (adjusted odds ratio (aOR) = 1.688, 95% confidence interval (CI): 1.495–1.905), the divorced (aOR = 1.430, 95%CI: 1.237–1.654), those traced through voluntary counselling and testing (VCT) (aOR = 1.707, 95%CI: 1.077–2.705) and those exposed to commercial sex (aOR = 1.437, 95%CI: 1.257–1.642) were more likely to receive tracing counselling. Among close contacts, those who were Han Chinese (aOR = 1.231, 95% CI: 1.012~1.498), married (aOR = 1.738, 95% CI: 1.339 ~ 2.255), had a high school education or higher (aOR = 2.809, 95% CI: 1.849~4.267) or were exposed to positive spouses or regular sex partners (aOR = 2.403, 95% CI: 1.741~3.317) were more likely to undergo HIV testing. A higher positive testing rate was observed among men (aOR = 2.608, 95% CI: 1.740~3.911), ethnic minorities (aOR = 1.645, 95% CI: 1.098~2.465), individuals aged 40–49 (aOR = 2.733, 95% CI: 1.497~4.990) and individuals exposed to positive spouses or regular partners (aOR = 2.215, 95% CI: 1.411~3.477).

**Conclusion:**

HIV recent infection testing is instrumental in enhancing the efficiency of contact tracing efforts. The variability in contact tracing uptake among diverse populations underscores the necessity for tailored strategies. These findings provide strong support for optimizing HIV prevention and control strategies, facilitating more precise disease management objectives.

## Introduction

Acquired immunodeficiency syndrome (AIDS) represents a significant global infectious disease, posing enduring threats to human health and social stability (1). Although advancements in HIV prevention and treatment have been achieved globally, new infections persist (2). A key challenge in combating HIV is the diversity and covert nature of its transmission routes; additionally, the asymptomatic nature of the incubation period in infected individuals complicates early detection and intervention efforts (3). Socioeconomic and cultural factors further influence the efficacy of prevention and control strategies. To curb the spread of HIV more effectively, it is necessary to pinpoint the transmission chain, promptly detect new infections and prevent further viral dissemination (4).

As an important prevention and control method, contact tracing helps to identify potentially infected groups and high-risk areas, discover newly infected individuals in a timely manner, and provide opportunities for early intervention and treatment by thoroughly exploring the source of infection and transmission routes of infected people (5). Contact tracing has been used in the control of transmitted diseases, such as tuberculosis and HIV (6, 7). During the Corona Virus Disease 2019 (COVID-19) pandemic, contact tracing played a crucial role in identifying and containing outbreaks, helping to mitigate the spread of the virus. Yunnan Province started HIV contact tracing pilot project since 2008 (8), and proposed the “HIV contact tracing” strategy in 2017. After practice and exploration, a complete theoretical system of tracing, counselling and testing has been formed and promoted throughout the province from 2018 (9, 10). To improve the relevant skills of staff, the Yunnan Center for Disease Control and Prevention has also conducted several rounds of training for provincial, prefectural and county staff at all levels to ensure the professionalism and effectiveness of contact tracing. Yunnan Province has achieved remarkable results in HIV contact tracing (9). First, contact tracing has successfully detected a large number of people living with HIV, providing opportunities for their early treatment and care. Secondly, the implementation of this strategy improves the efficiency and relevance of HIV/AIDS prevention and control work in the province, helping to better target key populations at higher risk and areas for intervention. In addition, by sharing its successful practical experience, Yunnan Province has provided a useful reference for HIV prevention and control work in other regions of the country (11).

However, many practical barriers remain. For example, different populations have different levels of uptake of contact tracing, which may be influenced by multiple factors, such as stigma/discrimination, social/economic isolation, privacy and confidentiality (5, 12, 13). Therefore, differentiated strategies based on the characteristics of different populations need to be developed to better promote the implementation of contact tracing. This study conducted an in-depth analysis of the implementation status of contact tracing in 2020. By comparing the differences in uptake of contact tracing among different populations, the factors influencing the effectiveness of contact tracing were explored. Meanwhile, this study also focused on the potential of recent infection testing in improving the effectiveness of contact tracing, with the aim of providing strong data support and practical experience for optimizing HIV prevention and control strategies and improving the effectiveness of prevention and control.

## Materials and methods

### HIV contact tracing

**Figure 1:**
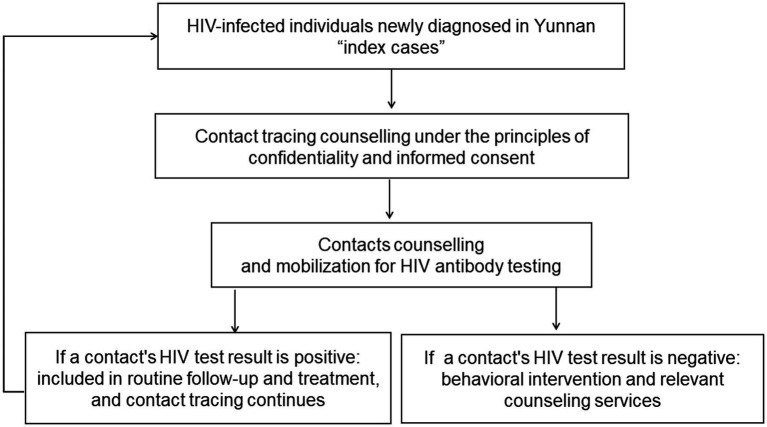
HIV contact tracing procedure in Yunnan province.

While ensuring confidentiality and fully respecting the privacy of both infected individuals and their close contacts, and with their informed consent, HIV contact tracing was implemented in 315 healthcare institutions in Yunnan Province during the first half of 2020. For individuals newly diagnosed with HIV, service providers conducted counseling to understand their high-risk behaviors and encourage them to disclose the names and detailed information of their sexual and injection drug-using partners. Close contacts were then informed and urged to undergo HIV testing. If a contact partner was newly diagnosed with HIV, they would be traced as a new HIV index. This process continued until no further contacts tested positive for HIV or no additional contacts were reported (Figure 1). All identified participants living with HIV were registered in the Chinese National Information System for HIV prevention and control (CNISAPC). They received regular follow-up and health care according to national guidelines, underwent testing to identify recent HIV infections, and their close contacts’ data were collected by the Yunnan Provincial Data Analysis Assistance System for HIV/AIDS Care. The written informed consents were obtained from the participants. This study was approved by the Ethical Review Committee of Yunnan Provincial Centre for Disease Control and Prevention (YNER 2020–4).

### Identification of recently infected HIV cases

Recently infected HIV cases were identified using the HIV-1 Limiting Antigen Avidity enzyme immunoassay (LAg-Avidity EIA, sourced from Maxim Biomedical, Inc., Rockville, USA). This assay helped distinguish between recent and long-term infections. The window period for the LAg-Avidity EIA is 130 days, which is the average length of time that individuals are classified as ‘recently infected’ by the LAg-Avidity EIA (14, 15). To minimize false positives, Recent Infection Testing Algorithms (RITAs) were employed. Before conducting the LAg-Avidity EIA, samples with CD4^+^ T cell counts under 200 cells/μl or showing signs of AIDS-defining illnesses were immediately categorized as long-term infections. Initially, all samples underwent a single-well preliminary screening test using the LAg-Avidity EIA. Samples yielding an ODn value exceeding 2.0 in this screening were deemed long-term infections. For samples with an ODn value of 2.0 or less, a three-well assay was conducted for verification. In this confirmation test, samples with an ODn value of 1.5 or below were labeled as recent infections.

### Statistical analysis

The SPSS 19.0 statistical analysis software (SPSS Inc., Chicago, IL) was utilized for statistical analyses. Initially, a univariate logistic analysis was executed, and variables exhibiting a *p*-value less than 0.10 were chosen for further multivariate logistic analysis, employing a backward unconditional approach. All statistical tests were conducted as two-tailed, and a p-value less than 0.05 was deemed statistically significant.

## Results

### Basic information of contact tracing

From January to June 2020, contact tracing was conducted for 5,695 newly diagnosed HIV cases in Yunnan Province. Of these, 1,612 cases (28.3%) received tracing counselling and provided contact information. Through these 1,612 individuals tested positive for HIV-1, 2,226 close contacts were successfully traced. Of these contacts, 910 (40.9%) underwent HIV testing, resulting in an antibody positivity rate of 17.8% (162/910). In addition, 132 cases were newly diagnosed, representing 14.5% of those tested.

### Recent infection and contact tracing

Among the 1,612 index cases, 11.3% (182/1612) were recently infected while 88.7% (1,430/1612) were long-term infected. From the recently and long-term infected index cases, 265 and 1961 close contacts were identified, respectively. On average, 1.46 ± 0.908 and 1.37 ± 0.875 close contacts were traced per recently and long-term infected individual, respectively, with no statistically significant difference between the two groups (*p* = 0.221).

Among the 265 close contacts provided by recently infected index cases, 94 underwent HIV antibody testing, with a testing rate of 35.5% (94/265). The positive detection rate was 26.6% (25/94), and the newly diagnosed positive rate was 22.3% (21/94). Among the 1961 close contacts provided by long-term infected index cases, 816 received HIV antibody testing, with a testing rate of 41.6% (816/1961). The positive detection rate was 16.8% (137/816), and the newly diagnosed positive rate was 13.6% (111/816). Both the positive detection rate and the newly diagnosed positive rate of close contacts undergoing testing provided by recently infected source infectors were significantly higher than those provided by long-term infected source infectors (Table 1).

**Table 1:** Contact tracing of index cases with different infection status.

Infection status	Total	Average number of contacts provided (mean ± SD)	*P*	Number of close contacts tested (%)	χ^2^	*P*	Number of HIV-positive persons (%)	χ^2^	*P*	Number of new HIV-positive persons (%)	χ^2^	*P*
Recent infection	182	1.46 ± 0.908 (265)	0.221	94(35.5)	3.641	0.056	25(26.6)	5.54	0.019	21(22.3)	5.189	0.023
Long-term infection	1,430	1.37 ± 0.875 (1961)		816 (41.6)			137 (16.8)			111 (13.6)		

### Factors associated with index cases acceptance of contact tracing counselling

The multivariate logistic analysis indicated that index cases belonging to other ethnic groups (adjusted odds ratio (aOR) = 1.688, 95% confidence interval (CI): 1.495–1.905), those who were divorced/widowed (aOR = 1.430, 95%CI: 1.237–1.654), those who were identified as infected through Voluntary Counselling and Testing (VCT) (aOR = 1.707, 95%CI: 1.077–2.705), and those whose contact mode was commercial sex (aOR = 1.437, 95%CI: 1.257–1.642) were more likely to accept contact tracing counselling and provide information about close contacts (Table 2).

**Table 2:** Factors associated with index cases’ acceptance of tracing counselling.

Demographic characteristics	Total	Number of people receiving tracing counselling (%)	Univariate analysis		Multivariate analysis	
			*P*	aOR (95% CI)	*P*	aOR (95% CI)
Total	5,695	1,612 (28.3)				
Sex						
Male	3,962	1,136 (28.7)	-	1.000		
Female	1733	476 (27.5)	0.353	0.942 (0.831–1.068)		
Ethnicity						
Han	3,463	836 (24.1)	-	1.000		
Others*	2,232	776 (34.8)	0.000	1.675 (1.490–1.882)	0.000	1.688 (1.495–1.905)
Age			0.187			
≥50	1849	489 (26.4)				
15–29	842	247 (29.3)	0.119	1.155 (0.964–1.383)		
30–39	1,418	418 (29.5)	0.055	1.163 (0.997–1.356)		
40–49	1,586	458 (28.9)	0.112	1.129 (0.972–1.312)		
Marital status			0.000		0.000	
Married	2,806	689 (24.6)	-	1.000		
Unmarried	1,480	456 (30.8)	0.000	1.368 (1.190–1.574)	0.036	1.175 (1.011–1.366)
Divorced/widowed	1,409	467 (33.1)	0.000	1.523 (1.324–1.753)	0.000	1.430 (1.237–1.654)
Education			0.555			
Illiteracy	880	238 (27.0)	-	1.000		
Primary school	2,476	702 (28.4)	0.458	1.067 (0.898–1.268)		
Junior middle school	1,597	470 (29.4)	0.209	1.125 (0.936–1.352)		
Senior middle school and above	741	202 (27.3)	0.923	1.011 (0.812–1.259)		
Occupation						
Farmer	4,315	1,249 (28.9)	-	1.000		
Others	1,380	363 (26.3)	0.058	0.876 (0.764–1.005)	0.379	0.936 (0.809–1.084)
Infection route			0.112			
Heterosexual contact	5,315	1,506 (28.3)	-	1.000		
Homosexual contact	295	90 (30.5)	0.421	1.110 (0.861–1.433)		
Intravenous drug use	85	16 (18.8)	0.056	0.586 (0.339–1.014)		
Sample source			0.000		0.000	
Spouse testing of positive individuals	225	34 (15.1)	-	1.000		
PITC	3,424	1,022 (29.8)	0.000	2.390 (1.648–3.468)	0.223	1.315 (0.847–2.041)
VCT	561	185 (33.0)	0.000	2.764 (1.843–4.144)	0.023	1.707 (1.077–2.705)
Marriage and pregnancy testing	253	78 (30.8)	0.000	2.504 (1.593–3.935)	0.063	1.612 (0.975–2.666)
Others	1,232	293 (23.8)	0.005	1.753 (1.190–2.583)	0.993	0.998 (0.633–1.574)
Contact mode			0.000		0.000	
Non-marital and non-commercial heterosexual contact	3,214	888 (27.6)	-	1.000		
Positive spousal/regular sexual partner contact	553	91 (16.5)	0.000	0.516 (0.407–0.654)	0.000	0.599 (0.451–0.796)
Commercial heterosexual sexual contact	1,548	527 (34.0)	0.000	1.352 (1.187–1.540)	0.000	1.437 (1.257–1.642)
Homosexual contact	295	90 (30.5)	0.291	1.150 (0.887–1.491)	0.150	1.232 (0.927–1.636)
Intravenous drug use exposure	85	16 (18.8)	0.075	0.607 (0.351–1.052)	0.123	0.645 (0.370–1.125)
Infection status						
Long-term infection	5,055	1,430 (28.3)	-	1.000		
Recent infection	640	182 (28.4)	0.937	1.007 (0.839–1.209)		

** Other ethnic groups include the following ethnicities: Bai, Blang, Buyi, Tibetan, Dai, De’ang, Dong, Dulong, Hani, Hui, Jingpo, Lahu, Lisu, Manchu, Mongolian, Miao, Mulao, Naxi, Nu, Pumi, Shui, Tujia, Wa, Yao, Yi and Zhuang.

### Factors associated with HIV antibody testing among close contacts

The multivariate logistic analysis (Table 3) showed that among the 2,226 close contacts, Han ethnicity individuals had a higher proportion of undergoing testing compared to other ethnic groups (aOR = 1.231, 95% CI: 1.012 ~ 1.498). Married/partnered individuals had a higher testing rate than unmarried individuals (aOR = 1.738, 95% CI: 1.339 ~ 2.255). Those with high school or higher education had a higher testing rate compared to illiterate individuals (aOR = 2.809, 95% CI: 1.849 ~ 4.267). Individuals who had sexual contact with positive spouses/regular partners had a higher testing rate than those who engaged in non-marital, non-commercial heterosexual behavior (aOR = 2.403, 95% CI: 1.741 ~ 3.317). However, individuals engaging in commercial sex (aOR = 0.460, 95% CI: 0.362 ~ 0.585) and intravenous drug users (aOR = 0.100, 95% CI: 0.013 ~ 0.766) had lower testing rates.

**Table 3:** Factors associated with HIV antibody testing among close contacts.

Demographic characteristics	Number of close contacts	Number of close contacts tested (%)	Univariate analysis		Multivariate analysis	
			P	OR (95% CI)	P	OR (95% CI)
Total	2,226	910 (40.9)				
Sex						
Female	1,401	536 (38.3)				
Male	825	374 (45.3)	0.001	1.338 (1.124–1.593)	0.292	0.889 (0.714–1.106)
Ethnicity						
Others	822	295 (35.9)				
Han	1,404	615 (43.8)	<0.001	1.392 (1.166–1.663)	**0.037**	**1.231 (1.012–1.498)**
Age			0.030		0.436	
15–30	580	213 (36.7)				
31–40	693	281 (40.5)	0.163	1.175 (0.937–1.475)	0.116	1.242 (0.948–1.627)
41–50	570	239 (41.9)	0.071	1.244 (0.982–1.577)	0.194	1.236 (0.898–1.700)
51-	383	177 (46.2)	0.003	1.480 (1.139–1.925)	0.187	1.280 (0.887–1.847)
Marital status			<0.001		<0.001	
Unmarried	809	292 (36.1)				
Married	754	380 (50.4)	<0.001	1.799 (1.469–2.203)	**<0.001**	**1.738 (1.339–2.255)**
Divorced/widowed	663	238 (35.9)	0.938	0.992 (0.801–1.228)	0.423	1.122 (0.847–1.487)
Education			<0.001		<0.001	
Illiteracy	265	97 (36.6)				
Primary school	882	310 (35.1)	0.664	0.939 (0.705–1.249)	0.733	0.949 (0.701–1.284)
Junior middle school	786	322 (41.0)	0.210	1.202 (0.902–1.602)	0.146	1.269 (0.920–1.750)
Senior middle school	293	181 (61.8)	<0.001	2.799 (1.985–3.946)	**<0.001**	**2.809 (1.849–4.267)**
Occupation						
Others	1,105	433 (39.2)				
Farmer	1,121	477 (42.6)	0.106	1.150 (0.971–1.361)		
Contact mode			<0.001		<0.001	
Non-marital and non-commercial heterosexual contact	1,270	541 (42.6)				
Positive spousal/regular sexual partner contact	214	145 (67.8)	<0.001	2.832 (2.082–3.851)	**<0.001**	**2.403 (1.741–3.317)**
Commercial heterosexual contact	566	135 (23.9)	<0.001	0.422 (0.338–0.528)	**<0.001**	**0.460 (0.362–0.585)**
Homosexual contact	159	88 (55.3)	0.002	1.670 (1.198–2.327)	0.379	1.204 (0.796–1.820)
Intravenous drug use	17	1 (5.9)	0.017	0.084 (0.011–0.637)	**0.027**	**0.100 (0.013–0.766)**
Infection status						
Long-term infection	265	94 (35.5)				
Recent infection	1961	816 (41.6)	0.057	1.296 (0.992–1.694)	0.100	1.267 (0.955–1.681)

### Factors associated with positive detection among close contacts undergoing testing

After multivariate analysis (Table 4), the following factors were significantly associated with a higher positive detection rate among close contacts who underwent testing: male (aOR = 2.608, 95% CI: 1.740~3.911), ethnic minorities (aOR = 1.645, 95% CI: 1.098~2.465), individuals aged 40–49 (aOR = 2.733, 95% CI: 1.497~4.990), and individuals with positive spouses/regular partners (aOR = 2.215, 95% CI: 1.411~3.477) (Table 3).

**Table 4:** Factors associated with positive detection among close contacts undergoing testing.

Demographic characteristics	Number of persons tested for HIV	Number of HIV-positive persons (%)	Univariate analysis		Multivariate analysis	
			P	OR (95% CI)	P	OR (95% CI)
Total	910	162 (17.8)				
Sex						
Female	536	65 (12.1)	–	1.000		
Male	374	97 (25.9)	<0.001	2.537 (1.793–3.592)	0.000	2.608 (1.740–3.911)
Ethnicity						
Han	615	96 (15.6)	–	1.000		
Others	295	66 (22.4)	0.013	1.558 (1.098–2.211)	0.016	1.645 (1.098–2.465)
Age			0.000		0.001	
15–29	213	25 (11.7)	–	1.000	–	1.000
30–39	281	36 (12.8)	0.719	1.105 (0.641–1.905)	0.717	1.115 (0.619–2.007)
40–49	239	59 (24.7)	0.001	2.465 (1.480–4.107)	0.001	2.733 (1.497–4.990)
50-	177	42 (23.7)	0.002	2.340 (1.360–4.024)	0.111	1.717 (0.883–3.339)
Marital status			0.359			
Unmarried	292	52 (17.8)	–	1.000		
Married	380	61 (16.1)	0.547	0.883 (0.588–1.325)		
Divorced/widowed	238	49 (20.6)	0.418	1.197 (0.775–1.847)		
Education			0.001		0.063	
Senior middle school and above	181	30 (16.6)	–	1.000	–	1.000
Illiteracy	97	29 (29.9)	0.011	2.147 (1.196–3.854)	0.162	1.740 (0.800–3.781)
Primary school	310	63 (20.3)	0.307	1.284 (0.795–2.074)	0.994	0.998 (0.527–1.889)
Junior middle school	322	40 (12.4)	0.198	0.714 (0.427–1.192)	0.319	0.736 (0.402–1.345)
Occupation						
Others	433	61 (14.1)	–	1.000		
Farmer	477	101 (21.2)	0.006	1.638 (1.156–2.321)	0.400	1.202 (0.783–1.845)
Contact mode			0.000		0.000	
Non-marital and non-commercial heterosexual contact	541	92 (17.0)	–	1.000		
Positive spousal/regular sexual partner contact	145	44 (30.3)	0.000	2.126 (1.398–3.233)	0.001	2.215 (1.411–3.477)
Commercial heterosexual sexual contact	135	7 (5.2)	0.001	0.267 (0.121–0.590)	0.007	0.321 (0.141–0.729)
Homosexual contact	88	18 (20.5)	0.430	1.255 (0.714–2.207)	0.553	1.241 (0.608–2.533)
Intravenous drug use exposure	1	1 (100.0)	1	7.884E+09 (−)	1.000	3.11E+09 (−)
Infection status						
Long-term infection	816	137 (16.8)	–	1.000		
Recent infection	94	25 (26.6)	0.020	1.796 (1.097–2.940)	0.057	1.680 (0.986–2.864)

## Discussion

This study analyzed data on contact tracing among individuals newly diagnosed with HIV in Yunnan Province from January to June 2020, with the objective of deepening our understanding of the factors that influence contact tracing effectiveness. Although only 28.3% of individuals living with HIV received contact tracing counselling, this group successfully traced a large number of close contacts, 40.9% of whom underwent HIV testing. This demonstrates the pivotal role of contact tracing in HIV prevention and control efforts. Furthermore, the screenings identified 132 new HIV infections, resulting in a new diagnosis positivity rate of 14.5%, which exceeds rates from VCT and provider-initiated counselling and testing (PITC) (9). These findings underscore the critical importance of HIV contact tracing in identifying new infections and preventing further spread of the virus. The high positivity rate observed among those screened highlights the necessity for sustained and expanded screening efforts (12).

Surveillance of recent HIV infections is crucial for evaluating trends in HIV prevalence and the efficacy of prevention and treatment interventions (16). When considering only the infection status of index cases, unreported positive cases were more likely to be found among close contacts of recently infected individuals. This could be attributed to the relatively brief interval between recent infection and exposure to the infectious source, potentially facilitating more precise recall and reporting of close contact details. However, a multifactorial analysis revealed no significant association between infection status and contact tracing acceptance or positive detection rates. It should be noted that undiagnosed HIV long-term infections may be the main source of continuous transmission (17). Consequently, contact tracing should integrate information from both recently and long-term infected individuals to comprehensively elucidate the dynamics of disease spread.

The acceptance of contact tracing involves sociological aspects, including culture, psychology, personal privacy, social responsibility, law and policy (18–20). After analyzing relevant factors, we found that individuals from other ethnic groups tended to be more receptive to HIV contact tracing and were more willing to share information about close contacts. Research showed that cultural differences influence health behavior and the effectiveness of health information (21). The cultural and social backgrounds of other ethnic groups may lead to a relatively open attitude towards their behavior, hoping to receive health information and decision support. Similarly, divorced or unmarried individuals were more likely to receive contact tracing counselling. Those not in a marital relationship might have fewer concerns about privacy when it comes to disclosing information about their contacts, as there may be less potential for conflict or judgment from a spouse (19). Furthermore, those who were diagnosed through VCT were more cooperative in tracing counselling. This cooperative attitude could stem from multiple factors. Firstly, actively seeking VCT indicates that individuals have developed a certain level of psychological preparedness, making them more willing to confront diagnostic results and participate in subsequent interventions (5). Secondly, the supportive environment provided by VCT services helps to build trust in the service, which lays the groundwork for subsequent sensitive contact tracing conversations (22). Furthermore, seeking testing often indicates a strong sense of health awareness and social responsibility. This intrinsic motivation aligns closely with the goal of contact tracing, which is to protect public health (23). Individuals infected through commercial sexual contact were also more willing to accept tracing counselling, possibly because they had a clear understanding of their infection routes and were thus more willing to provide information.

The proportion of close contacts undergoing HIV testing was influenced by various factors. Firstly, the proportion of Han ethnicity undergoing HIV testing was significantly higher than that of other ethnic groups. This may be due to the easier access of the Han population to relevant health promotion and education, thereby increasing their awareness and uptake of HIV testing (24). Secondly, marital status also significantly affects the proportion of testing. A previous study showed spouses and long-term heterosexual or homosexual partners were willing to receive HIV testing (25). In terms of educational level, groups with high school or higher education have a higher proportion of testing. This indicates that higher education levels enhance individuals’ cognition and emphasis on health issues, thereby increasing their willingness to uptake of HIV testing (26, 27). Individuals exposed to HIV-positive spouses or regular sexual partners have a significantly higher proportion of testing than other groups, possibly because they face a more direct risk of HIV infection and thus attach greater importance to testing. In China, follow-up of sero-discordant couples and testing of sero-negative partners is required (28). A recent study also found that sero-negative partners among sero-discordant couples (SNPs) were more likely to be frequent HIV testers (AOR = 4.02, 95% CI: 2.78–5.83) (29). Conversely, individuals engaged in commercial sex and intravenous drug users had a lower proportion of testing, which may be related to their lifestyle, social marginalization, and neglect of health issues (30). A relevant study also suggested that willingness to receive HIV testing was low among casual and commercial sex partners of ‘index cases’ (25).

Overall, the testing rate for close contacts was insufficient, which limited the effectiveness of contact tracing to some extent. Beyond the aforementioned factors (such as ethnicity, marital status and level of education), there may be multiple social and structural barriers. Firstly, stigmatization and social discrimination may cause contacts to refuse testing out of fear of identity exposure (31). Secondly, highly mobile populations (such as migrant workers or sex workers) are difficult to track and follow up with. Thirdly, despite stringent privacy protections, contacts may still be concerned about leaks of their personal information. Finally, access barriers must be addressed, including inconvenient testing site locations, scheduling conflicts with work, and misunderstandings about testing procedures and costs. Increasing testing coverage is crucial for breaking HIV transmission chains, as undetected infections continue to spread the virus (32). Future interventions must therefore develop more targeted, culturally sensitive and accessible strategies to address these barriers. This includes promoting self-testing, expanding non-site-based testing services and running anti-stigma campaigns to encourage more people at risk of infection to get tested.

The positive detection rate among close contacts undergoing testing was influenced by various factors, including gender, ethnicity, age, and relationship with the persons living with HIV. Male close contacts were more likely to test positive for HIV infection, which may be related to their social and sexual behaviors. In some cultures, men may be more inclined to engage in high-risk sexual behaviors, such as having multiple sexual partners or engaging in commercial sexual activity without adequate protection (33). Men may not be as aware of or value sexual health issues as women, leading them to be more likely to overlook HIV prevention and testing. Although the proportion of close contacts from other ethnic groups undergoing testing is not high, their positive detection rate was higher than that of the Han ethnicity. This suggested, to some extent, that they may face barriers to information access, preventing them from fully understanding HIV transmission routes, prevention measures, and testing methods. Due to a lack of relevant knowledge, they may find it difficult to make clear, objective judgements about the risk level of their own behaviors compared to groups with greater access to information. This can result in them underestimating the actual risk of infection or lacking clarity on how to take effective protective measures. The positive detection rate was higher among individuals aged 40–49 than among those aged 15–29. This suggests that middle-aged individuals may be at risk of infection due to unique factors, and that future research should focus on the underlying behavioral or sociological drivers, such as specific sexual networks or preventive awareness. The positive detection rate was also significantly higher among those with HIV-positive spouses/regular sexual partners, indicating a need to strengthen the management of infected individuals to prevent transmission between spouses. Health education should be enhanced to raise awareness of prevention and testing, while providing necessary psychological support and social assistance (34, 35).

Of the close contacts who tested positive, 30 had previously been confirmed as HIV-positive. This suggested that they may still pose a risk of further transmission. It is important to improve our understanding of the treatment status of individuals at risk of transmission, and to ensure they are effectively engaged in care. This phenomenon also highlights a weak link in the ‘treatment linkage and sustained care’ process between ‘diagnosis’ and ‘viral suppression’. Notably, Yunnan Province, a key region for HIV prevention and control in China, achieved the 2020 UNAIDS 90–90-90 interim target (36). This achievement signifies significant progress in expanding testing and treatment coverage within Yunnan’s prevention and control system. However, the issues revealed by this study clearly indicate that advancing from ‘90–90-90’ to the more challenging ‘95–95-95’ target will require maximum effort to be put into prevention (37, 38). The core challenge has shifted from expanding coverage to improving the quality and depth of services. Building upon high diagnosis rates, future efforts must priorities strengthening long-term, high-quality follow-up management for diagnosed individuals. This will ensure sustained treatment adherence and achieve durable virological suppression.

The findings of this study suggest that different populations should be subject to different contact tracing strategies to enhance the efficiency and effectiveness of tracing, testing and consultation efforts. Firstly, as close contacts identified through recent index cases exhibit significantly higher positive detection and recent infection rates, public health resources should be prioritized for this group. This involves conducting faster and more thorough tracing consultations to ensure their contacts undergo testing. Secondly, communication and mobilization methods should be tailored to specific groups. In light of the finding that ethnic minority index cases exhibit a higher willingness to seek counselling, but lower contact testing rates and higher positivity rates, culturally sensitive communication strategies should be adopted. This could include training community health workers from ethnic minority backgrounds or using materials in native languages to build trust. Conversely, for contacts with higher education levels and greater willingness to be tested, digital tools can be used to enhance efficiency. Simultaneously, risk assessment based on exposure pathways is crucial. For individuals with high testing and positivity rates, such as HIV-positive spouses or regular sexual partners, comprehensive contact tracing and immediate intervention are essential. Conversely, a shift to more protective, low-threshold service models is required for commercial sex contacts and intravenous drug users with extremely low testing rates. This includes integrating testing services with community outreach efforts and promoting anonymous self-testing to overcome barriers to participation. In summary, the key to implementing a ‘targeted approach’ is to adopt communication strategies and service delivery methods that are tailored to the sociocultural characteristics and risk profiles of different populations. This will enhance the overall effectiveness of contact tracing.

This study has several limitations. Firstly, the findings were derived from a specific cohort in Yunnan Province, China, which may restrict how widely the results can be applied to other populations or regions with different healthcare infrastructures and epidemic profiles. Secondly, as this was an observational study, it is subject to potential unmeasured confounders that could influence the outcomes of contact tracing. These include socioeconomic factors, stigma and individuals’ mobility, all of which were not fully accounted for. Thirdly, the evaluation did not include an assessment of the resources available for contact tracing, a critical factor in determining its effectiveness. Future studies should incorporate rigorous evaluations of resource allocation and broader contextual factors.

## Conclusion

This study undertook a comprehensive analysis of the status of tracing counselling implementation in Yunnan Province. Our findings suggested that acceptance of counselling and testing was associated with a variety of factors and varies significantly across different populations. The factors influencing close contact testing and positive detection rates underscored the importance of tailored approaches to advance tracing counselling and testing efforts. Additionally, we noted that detecting recent infections played a crucial role in enhancing tracing efficiency, and its utilization in practical applications deserved greater emphasis. These valuable insights offer robust data-driven support for refining HIV prevention and control measures, thereby boosting the overall prevention and control effectiveness.

## Data Availability

The original contributions presented in the study are included in the article. Further inquiries can be directed to the corresponding author.
